# NeuroSense: A non-invasive and configurable somatosensory stimulator with OPENVIBE communication

**DOI:** 10.1016/j.ohx.2024.e00529

**Published:** 2024-04-18

**Authors:** Erick A. Gonzalez-Rodriguez, Luis Kevin Cepeda-Zapata, Angel Antonio Rivas-Silva, Vania G. Martinez-Gonzalez, Luz Maria Alonso-Valerdi, David Isaac Ibarra-Zarate

**Affiliations:** a Autonomous University of Nuevo Leon, Pedro de Alba S/N, Niños Héroes, Ciudad Universitaria, 66455 San Nicolás de los Garza, N.L., Mexico; b Instituto Tecnológico de Estudios Superiores de Monterrey, Av. Eugenio Garza Sada 2501 Sur, Tecnológico, 64849 Monterrey, N.L., Mexico

**Keywords:** Touch, Air, Vibration, Stimulation, Somatosensory evoked potentials, Tactile sensations

## Abstract

Understanding the somatosensory system and its abnormalities requires the development of devices that can accurately stimulate the human skin. New methods for assessing the somatosensory system can enhance the diagnosis, treatments, and prognosis for individuals with somatosensory impairments. Therefore, the design of NeuroSense, a tactile stimulator that evokes three types of daily life sensations (touch, air and vibration) is described in this work. The prototype aims to evoke quantitative assessments to evaluate the functionality of the somatosensory system and its abnormal conditions that affect the quality of life. In addition, the device has proven to have varying intensities and onset latencies that produces somatosensory evoked potentials and energy desynchronization on somatosensory cortex.

## Highlights

• A system composed out of four modules (one master module and three submodules) that evokes three types of stimuli: touch, air, and vibration.
• The system works with python scripts and OpenViBE open software via serial port, and radiofrequency telecommunication for connection between modules.
• Modules are portable and are easy to assemble.
• Air and Touch module had 500 ms delay between the stimulus cue and initialization of actuators. Vibration had around 150 a ms delay.

## Graphical abstract

**Figure:**
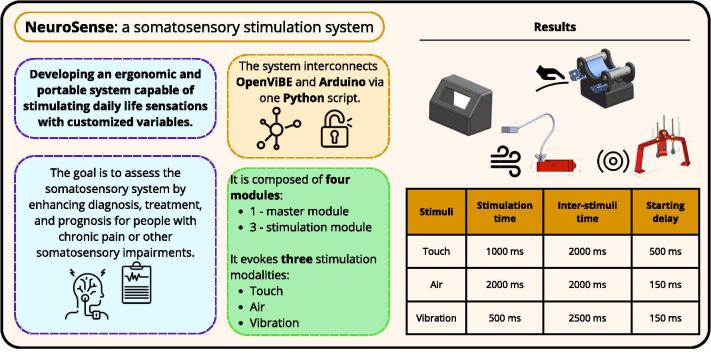

Specifications Table

**Table:** 

Hardware name	NeuroSense
Subject area	• Engineering and Material Science • Neuroscience • Educational Tools and Open-Source Alternatives to Existing Infrastructure
Hardware type	• Field measurements and sensors • Electrical engineering and computer science • Mechanical engineering and materials science
Open Source License	Creative Commons Attribution 4.0 International licence.
Cost of Hardware	530.60 USD
Source File Repository	https://doi.org/10.17632/h784hj9s83.1

## 1 Hardware in context

### 1.1 Background

#### 1.1.1 Somatosensory system physiology

The somatosensory system communicates about the current position of our body, and the properties of the environment to the central nervous system (CNS) [1]. It is composed of multiple mechanical receptors whose primary function is to perceive tactile sensations, skin stretches, environmental temperature measurement, pain detection, and proprioception (kinesthesia).

There are different mechanoreceptors found within the skin that can detect touch, pressure, and vibration by transducing physical stimuli [2]**.** These can be classified based on the intensity at which the action potential occurs, and the duration of the neural activity. For small-innocuous stimuli, mechanoreceptors are referred to as Low Threshold Mechanoreceptors (LTMR), while for strong-noxious ones, they are named High Threshold Mechanoreceptors (HTMR). Regarding the neural firing, they could be Rapid Adapting (RA), which fire action potentials are only at the onset and sometimes at the end of a step indentation; or Slow Adapting (SA), which exhibits spikes throughout the duration of a sustained step indentation.

Finally, the mechanoreceptors vary according to the individual’s skin, but they are normally distributed in the glabrous skin (palms and soles) and the hairy skin [2], [3]. Table 1 summarizes some of the different mechanoreceptors in the body alongside their fibers and optimal stimuli.

**Table 1:** Mechanoreceptors and associated fibers.

Physiological subtype	Associated fiber	Skin type	End organ/ending type	Optimal stimulus	NeuroSense sensations
SAI-LTMR	Aβ (16–96 m/s)	Glabrous/Hairy	Merkel cell	Indentation	−
RAI-LTMR	Aβ (26–91 m/s)	Glabrous	Meissner corpuscle	Indentation and vibration	Vibration
RAII-LTMR	Aβ (30–90 m/s)	Glabrous/Hairy	Pacinian corpuscle	Vibration	Vibration
Aδ-LTMR	Aβ (5–30 m/s)	Hairy	Longitudinal lanceolate ending	Direction-selective hair deflection, skin stroke and indentation	Air/Caress
C-LTMR	C (0.2–2 m/s)	Hairy	Longitudinal lanceolate ending	Hair deflection, skin stroke and indentation	Air/Caress
HTMR	Aβ/Aδ/C (0.5–100 m/s)	Glabrous/Hairy	Free nerve ending and circumferential ending	High-force indentation and hair pull	−

#### 1.1.2 Event related potentials

Electroencephalography (EEG) is a non-invasive neuroimaging technique of high temporal resolution. EEG signals yield comprehensive neural information when linked to specific events related to a stimulus [4].

One common method that uses EEG to assess somatosensory functionality is somatosensory event potentials (SEP), which arises in the CNS after sensory receptors are activated beyond their resting threshold by tactile sensations [1]. In this matter, tactile sensations referred to the perception of mechanical stimuli impacting the skin [1].

In clinical settings, SEPs are usually limited to electrical or laser stimulation [5], [6]**.** Therefore, for clarity in this article, authors will refer as SEP to cortical responses evoked by these types of stimulation only. The term evoked potential (EP) will also be used but referred to those evoked by any sensory stimuli [1], [6]. And finally, tactile evoked potential (TEP) for the ones evoked by stimulation on the skin (e.g., caress, vibration, touch, pressure, or indentation).

EPs can provide information about the amplitude, latency, polarity, sensitivity, and topographical distribution of the brain responses when stimulated [1]**.** These can be of short oscillations (<50 ms), which reflect the exogenous processing of the stimuli, or late oscillation (>100 ms), which reflect the interpretation of such stimuli. The first ones are commonly used in clinical settings to study the transmission from the nerves endings to the brain**,** while the second ones are used for studying the cognitive load that the person requires to understand and interpret the stimuli [1].

#### 1.1.3 Other Hardware devices

Quantitative Sensory Testing (QST) represents a nonintrusive method for evaluating and measuring the functionality of sensory nerves [7]**.** The Peripheral Neuropathy Association describes QSTs as methods that quantify the strength of stimuli required to elicit particular sensory experiences [8]**.**

Many QST devices have been developed with the idea of observing objective measures from the brain after stimuli. However, it must be mentioned that there are QST that rely solely on the perception of the subject rather than a quantitative physiological response such as EPs. Table 2 describes some devices and their characteristics.

**Table 2:** Review of state-of-the-art devices that evoke distinct types of EPs.

Type of Stimuli	Study	Control method		Control Variables	Description
Identation/Tactile	[9]	Arduino		Stimulation pattern and frequency	Controls stimulation patterns through an Arduino.
	[10]	Data Acquisition Card and LabView	Intensity, duration, and pattern of the stimuli	The device is placed on the fingertip and secured with Velcro. A pneumatic projectile moves and stimulates the skin.	
Vibration/Tactile	[11]	OpenViBE	Stimulation times	Two modules: (1) Vibrotactile interface. (2) Signal acquisition module.	
				The system stimulates two points of the face and both middle fingers.	
	[12]	ATmega128 and E-Prime software	Frequency (20–400 Hz at 40 levels) and intensity (0–7 V, at 256 levels)	A planar coil actuator generates vibrations through interaction with the static magnetic field of the MR scanner.	
	[13]	Computer assisted	Frequency and amplitude of tactile stimuli	Low-cost and compact stimulator. It delivers a range of tactile stimuli frequencies and amplitudes.	
Pneumatics	[14]	ATmega128 and E-Prime software	Air-puffs and suction pressure (ranges 2–6 psi in 1 psi steps)	Complete pneumatic system that stimulates fingertips.	
	[15]	Computer and pneumatic controller	Pressure (30 and 40 psi)	Solenoid air valves stimulate with air on the face and fingertips.	
Touch, vibration, pin-prick, heat-, cold-, warmth	[16]	Manual, computer assisted		Depends on the device.	Protocol for assessing most fiber subtypes through several QST and EEG.

### 1.2 Problem statement

Rating scales are commonly employed to evaluate peripheral neuropathic pain sensations in individuals, yet these scales lack objective measures. The *McGill Pain Questionnaire (MPQ)* serves as an illustrative example of such a scale, which assesses pain in patients undergoing chemotherapy [17] or experiencing neuropathic pain allodynia [18]. A significant challenge in epidemiological studies of allodynia pain is the subjective nature of pain symptoms, which hinders the reliability of validation studies. The prevalence of allodynia in neuropathic pain is also challenging to determine due to this subjectivity. The detection of evoked phenomena is influenced not only by the patient cohort but also by the criteria and methodologies applied in evaluating these responses. Relying solely on subjective questionnaires does not fully capture the individual's syndrome or provide insights into the brain's processing of sensations [19], [20]. Consequently, there is a need for standardized quantitative tools to achieve objective measurements of these complex human sensations.

Moreover, the scarcity of standardization in devices that evoke TEPs makes it difficult to compare results between different studies, despite using similar stimuli (as seen in Table 2). For instance, pneumatic devices varied their pressure levels among 30–40 psi [15], 2 or 6 psi [14], or 5 bar [21]. And other ones, such as vibrotactile-based devices, do not mention parameters [9], or work with small voltage values (3.7 V) at high velocity (1000 RPM) [16].

Furthermore, the stimuli type must be considered too. Some works stimulate with tools that can be either harmful like laser [22], [23] or invasive for the users [11], [15]. Other works use contact-heat stimulation devices [23], which may be less painful than laser-based ones and result in less evident lesions. However, their use is limited to research purposes [23]. Hence, finding prototypes that reproduce daily life sensations is difficult, although they could be useful for analyzing the somatosensory system under day-to-day conditions.

### 1.3 Justication

This work introduces NeuroSense, a portable easy to assemble tactile stimulator device that connects with open-source applications for studying the somatosensory system. The proposed device is innocuous and is not expected to cause pain or to activate HTMR of any type. Instead, NeuroSense reproduces daily life sensations, such as airflow, vibration, and caress, by stimulating specific mechanoreceptors and nerve fibers (these can be seen in Table 1). This makes it suitable for assessing complex pain process such as that caused by allodynia in neuropathic pain patients [19].

Furthermore, NeuroSense proposes to standardize stimulation parameters such as stimulation time, inter-stimuli time, and physical stimulation variables. It can be programmed with protocols that either can increase gradually the intensity of the stimuli or ser parameters to allow replicable results that could be compared among different investigations. Besides, our prototype can link open-source applications. Software tools such as OpenViBE, Miniconda, and Arduino are used to integrate the system in a complete open-source interface. This would allow investigators and clinicians to carry out real-time studies easily with tools that are in the reach of everyone and without external costs.

### 1.4 Aim

The aim of this project is, therefore, to develop a prototype that can stimulate the somatosensory system by using vibration, pressure, and air, while also being able to control intensity, duration, and number of repetitions of the stimuli being used. In addition, this prototype is controlled via OpenViBE, one of the most widely and commonly used platforms to develop neuroscience based experimental paradigms. Finally, this article pursues to provide all the necessary details to be replicated by anyone who might be interested.

## 2 Hardware description

### 2.1 OpenViBE-Python-Arduino connection

**Fig. 1:**
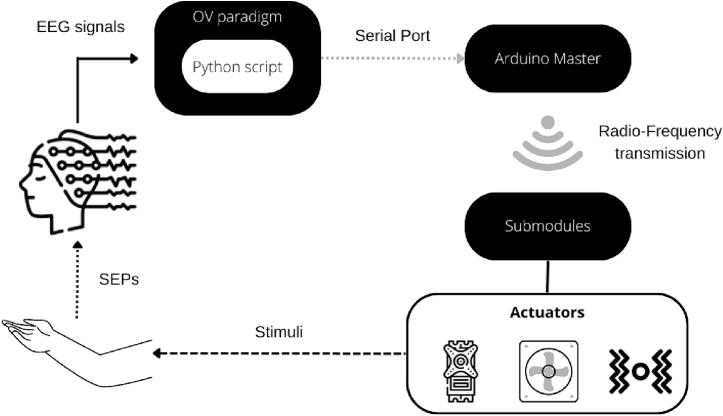
General structure and connection of the system.

The main characteristic of **NeuroSense** is its property to connect all modules to OpenViBE (OV). It is based on serial communication between OV and Arduino using a Python script as an intermediary. Fig. 1 shows the connection between each part of the prototype (hardware and software). The system works as follows:

1. The paradigm starts and sends stimulation labels.
2. The Python script within the OV paradigm (“*run command*” block) reads and interprets the labels, and sends them as characters via serial communication protocol to Arduino.
3. The Master module’s Arduino program reads each character and determines which submodule the label belongs to. Then, the label is sent to the submodule via radio frequency communication.
4. Once the submodule receives the label, the actuators are activated.

### 2.2 Hardware

NeuroSense hardware communicates via radio frequency protocol. The device is composed of four modules (Master and three submodules), which all have one nRF24L01 radio frequency driver connected to an Arduino NANO microcontroller. The Master module is connected to the computer and OV, while the others are controlled by this module.

#### 2.2.1 Master module (MASTM)

**Fig. 2:**
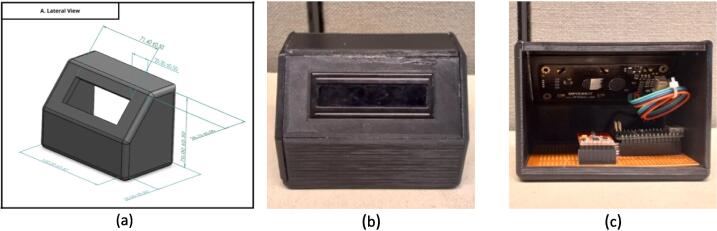
MASTM. In figure (a), the 3D model of the module with dimensions in “mm”. (b) Shows the front face of the module with the LCD screen, and (c) allows to see the circuit inside the box.

MASTM works as a transmitter that sends OV’s stimulation labels to the three submodules (Air, Touch and Vibration). It has one RGB Liquid Cristal Display (LCD) to visualize the transmission status while showing the code label sent. The circuit is covered by a 3D printed box and assembled with adhesive. Its dimensions are 11 cm x 6.5 cm x 8 cm (length x width x height), which makes it portable and easy to handle (module in Fig. 2).

#### 2.2.2 Air module (AIRM)

AIRM uses a 12 VDC fan as the actuator that can be positioned over different points over the arm due to a flexible cupper arm attached to its base. The module’s intensity level is translated to analog output using a PWM Arduino pin (5 VDC as the maximum possible) that energizes one IRFZ44N MOSFET that lets the voltage pass from a Step-Up Voltage Regulator of 4–12 VDC to the fan. Hence, the fan speed and the airflow are regulated.

**Fig. 3:**
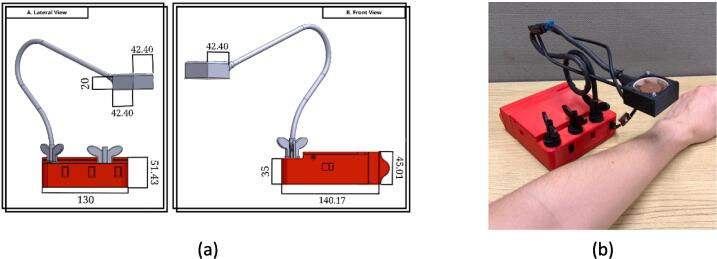
AIRM. Figure (a) shows the complete model with dimensions in “mm”, and (b) the correct positioning of the arm when the module is used.

The dimensions of this module vary depending on the extension of the cupper arm. The standard position with the arm folded (in Fig. 3) has around 25 cm x 5 cm x 18 cm of dimensions. Below, a base works as counter-weight to the fan.

#### 2.2.3 Touch module (TOUM)

**Fig. 4:**
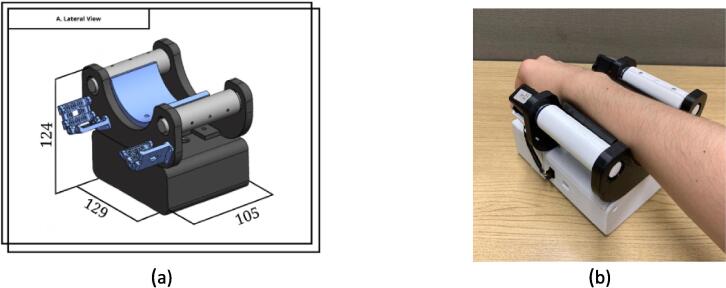
TOUM. Figure (a) shows the 3D CAD assembled model (dimensions in “mm”), and (b) the module with the arm placed on it.

This module evokes the sensation of a caress on the arm. It has dimensions of 16 cm x 15 cm x 14 cm, allowing secure transportation. The module’s final design can be seen in Fig. 4.

It employs two 12 VDC Dynamixel AX-12a servo-motors that tension a piece of cloth to a specific tightness according to the Arduino program, pressing the arm against the plate. A SN74LS241N tri-state buffer was used as it allows the transmission and reception of information to the servomotors. A switch is connected to interrupt the energy from a Li-Po 11.1 VDC optimal battery.

Underneath the plate, a TAL220B load cell is placed to gauge the pressure being applied by the cloth in the tensioning step. A HX711 weight conversion module was then used to convert the load cell’s data for input into the Arduino. This process is done to achieve the desired tension by adjusting both servo-motors to the necessary angle and speed.

#### 2.2.4 Vibration module (VIBM)

**Fig. 5:**
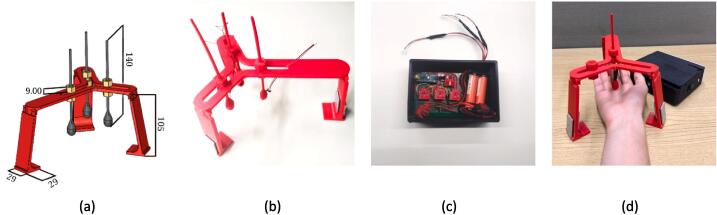
VIBM. In figure (a) the 3D model of the module with dimensions, (b) shows the tripoded structure with ERM motors inside the sticks, (c) has the circuitry with the batteries and the cables that will connect to the ERM motors, and (d) the arm placed below the structure ready to be used.

The VIBM comprises two main components: a box that houses the circuitry and a versatile structure designed to stimulate at different points. The dimensions of the box is 16 x 11 x 6 cm, whereas the structure measures approximately 20.5 cm x 16 cm x 10.5 cm. This box features two openings: one allows for the connection of the drivers to the Eccentric Rotating Mass (ERM) motors located in the stimulation structure; the other facilitates the connecting of the Arduino to a laptop. The tripoded stimulation structure manages three sticks with ERM motors within them. This design enables the precise positioning of the motors over specific hand stimulation points. Fig. 5 illustrates the final design of this module.

After the Arduino processes the stimulation codes, it signals the DRV2605L haptic drivers. The driver controls the ERM vibration motor according to the programmed sequence. Additionally, a voltage regulator powers Arduino, allowing its wireless usage.

## 3 Design files summary

In Table 3, the files used for all modules are listed.

• **3D design files (.sldprt):** SolidWorks 2017 and 2019′s files of all parts for each module.
• **3D printing files (.stl):** Stereolithography file that can be opened on Ultimaker Cura Software or any other 3D printing software that supports it.
• **Python script (.py):** Python script file that links the OV environment with Arduino. It can be ran in the command window or, preferably, alongside the OV paradigm (using “*run command*” block).
• **OpenViBE paradigms (.xml)***:* OV files of the paradigms that contain function blocks used to save EEG signals and to export information like time and stimulation codes.
• **Paradigms’ codes (.lua)**: Lua file that defines and sends stimulation codes. These files must be imported to their respective OV paradigm.
• **Keyboard codes for OV (.txt):** text file of 2–3 lines that indicates the stimulation codes related to keyboard entries. This file must be uploaded to all OV paradigms, as it indicates the start and finish of the experiment.
• **Arduino codes (.ino):** Arduino files from each module. They contain comments and clarifications describing functions, actions, variables, libraries used, etc.
• **Libraries**: a.zip file which contains all libraries used for the Arduino scripts.
• **PCB schematics (.grbr)**: gerber files ready for printing circuit boards.

**Table 3:** **All protype’s files**. In this table, a general label for all OV paradigms, Lua files, Python sketch, Arduino codes and design files is mentioned with its type of file. All of them are added to the database generated with this article.

Design filename	File type	Open-source license	Location of the file
3D design files	.sldprt	Creative Commons − Attribution 4.0 International	Dataset link: *https://doi.org/10.17632/h784hj9s83.1*
3D printing files	.stl		
Python script	.py		
OV paradigms	.xml		
Paradigms’ codes	.lua		
Keyboard codes for OV	.txt		
Arduino codes	.ino		
Libraries	.zip		
PCB schematics	.grbr		

## 4 Bill of material

A table of materials was produced to indicate which electronic components are needed to build each module. Some items can be easily found at any electronic store, while other components, such as the servo-motors or the RF module, had to be pre-ordered. For each material, a link to the online store is provided.

Readers may be concerned about the raw material required for 3D printing. On the able, it is recommended to use 1.75 mm PLA filament. Although, the use of other material can be possible by taking the correct printing considerations.

The complete list of materials can be found in Mendeley database at https://doi.org/10.17632/h784hj9s83.1.

## 5 Build instructions

Firstly, read the instructions and ensure you have all.stl files printed before the building process. Some steps will need tools such as screwdriver, nuts, or glue, depending on the case.

**Fig. 6:**
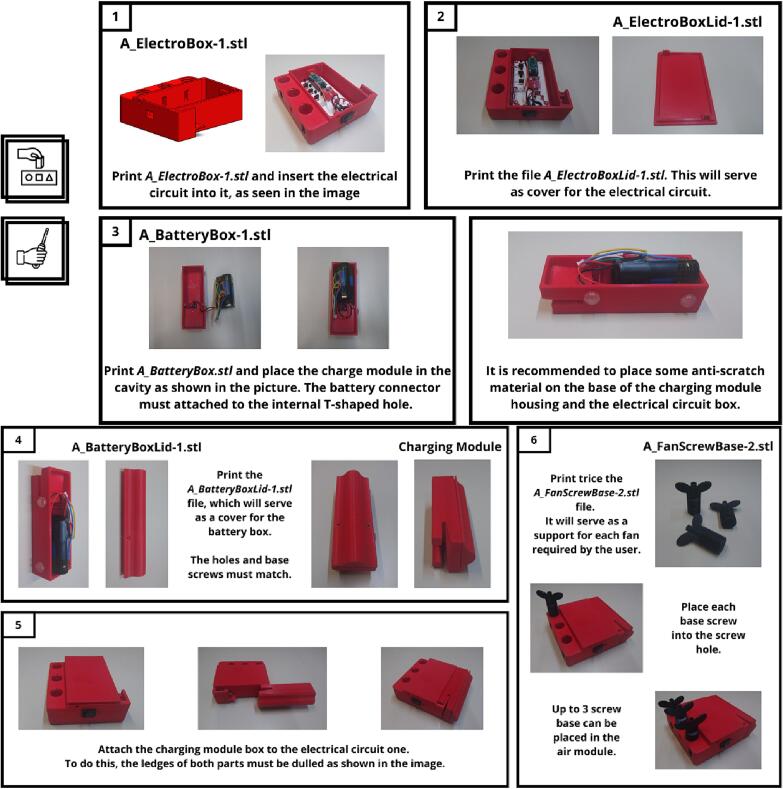

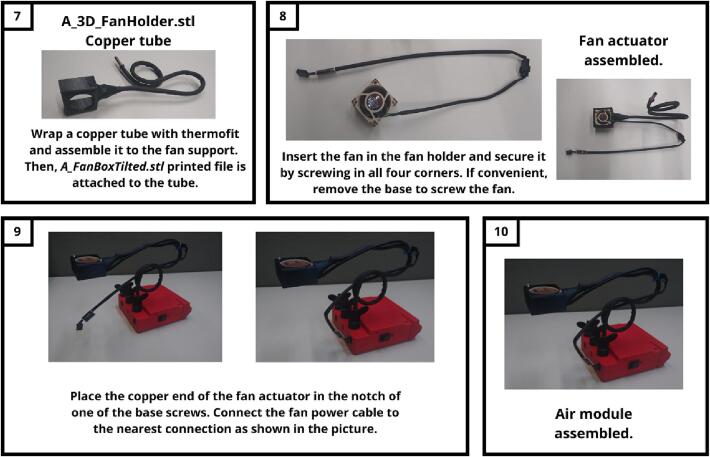
Air module construction instructions. This module makes an exception to its materials, as it requires a non-printed piece which corresponds to a flexible copper tube covered with a thermofit, which is used to avoid tube deformation due to frequent managing.

**Fig. 7:**
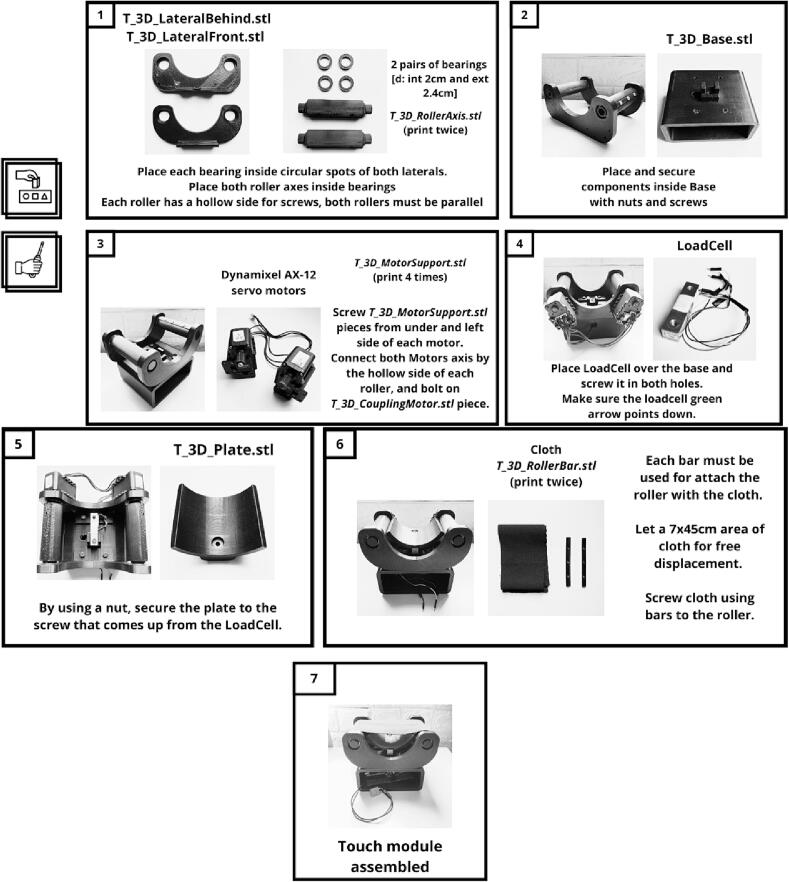
Touch module building instructions.

**Fig. 8:**
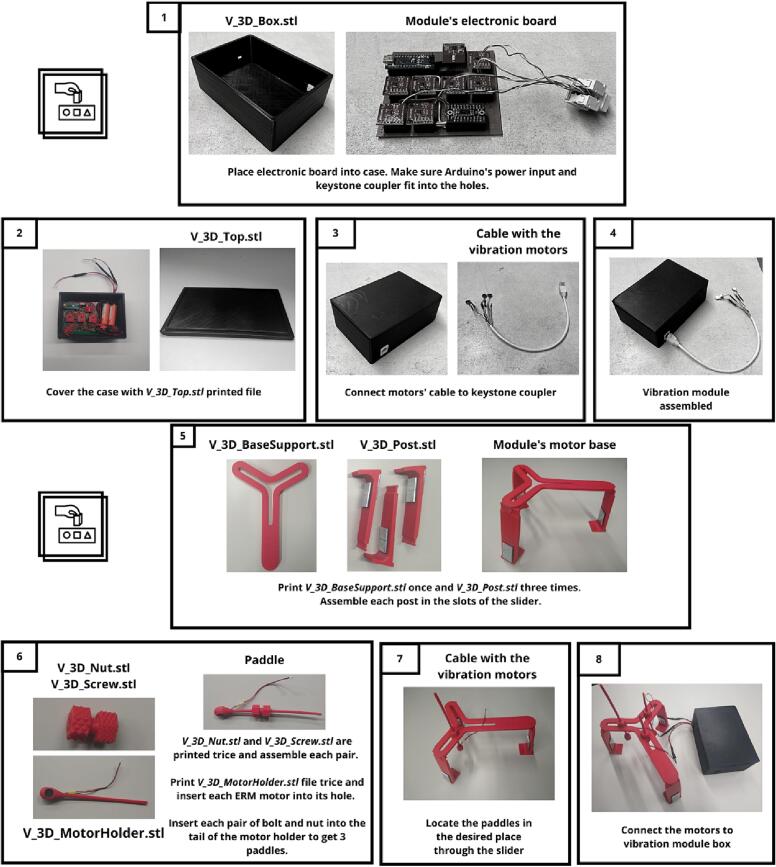
Vibration module assembly instructions.

The following figures explain how to assemble each module step by step: AIRM (Fig. 6), TOUM (Fig. 7), and VIBM (Fig. 8). The MASTM can be printed as a single piece without further instructions. The icons outside of the instructions indicate which tools are needed or if assembling is enough. Each step is described inside a rectangle. On its left, side is the result of the previous step, and on its right is the new part and its name on the top. Instructions for every step are provided at the bottom.

## 6 Operation instructions

**Fig. 9:**
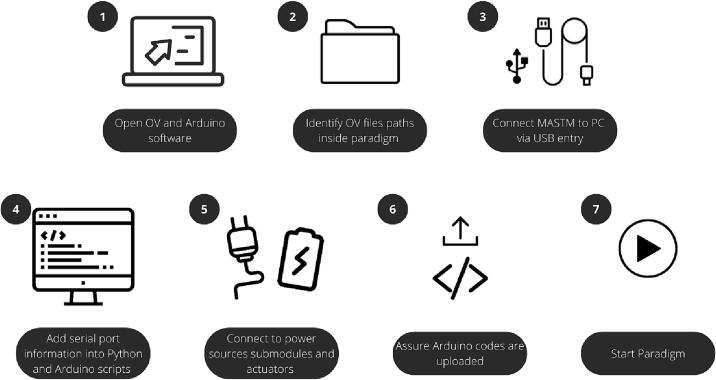
General procedure. Steps to follow before starting the paradigm.

The general procedure to use the devices is shown on Fig. 9. It is important to also consider the following points to use the device properly:

**Pre-installed software**.

• OpenViBE (version 3.0.0) with “*run command*” block (supports Python version 3.7).
• Arduino.
• Just in case, use any script editor (such as Visual Studio or SciTe) with python and lua languages support.

**Code considerations:**

• Arduino libraries to download (in the database): DFrobot_LCD, Dynamixel_Serial, Haptic_Motor_Drive, SparkFun_Haptic_Motor_Driver_Arduino_Library and HX711_ADC, RF24.
• For Python script it is necessary to install libraries *pylsl* and *pyserial*.

**Hardware considerations**.

• For AIRM and TOUM, it is not recommended to use the same power source for both Arduino and actuators. Or use voltage regulator of maximum 5 V.
• For TOUM, it is necessary to first calibrate the load cell before starting paradigm. The load cell library contains an example code for this task.
• Make sure that the cloth in the TOUM is not initially tensed.

## 7 Validation and characterization

### 7.1 Measurements

A paradigm was specifically designed to validate how consistent the devices are in terms of onset latency and intensity differentiability. The paradigm consisted of a continuous train of 120 triggers (30 for each intensity) for TOUM, VIBM and AIRM. For VIBM, a stimulation of 500 ms was used with a resting time of 2500 ms. TOUM used a 1000 ms stimulation interval and 2000 ms rest interval. Lastly, AIRM used 2000 ms for stimulation and 2000 ms for rest. The aim was to record each intensity value as a continuous response from the start (*t_1_*) to the end of stimulus (*t_2_*) and that each intensity has a higher value than the previous one.

**Fig. 10:**
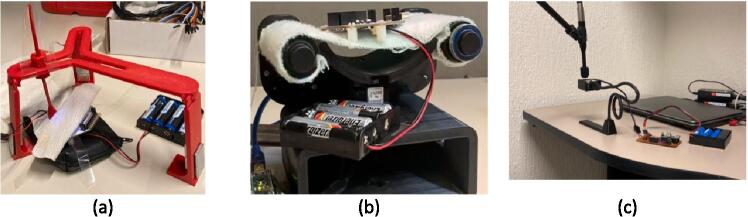
OpenBCI with a) VIBM and b) TOUM and c) DBX RTA-M with AIRM.

To measure the magnitude of the intensities in TOUM and VIBM, the LIS3DH accelerometer of the OpenBCI Cython module was employed. This accelerometer was positioned beneath the VIBM actuator, as shown in Fig. 10(a). The vibration caused by the ERM's rotation was captured by the accelerometer. Once the ERM starts rotating, the vibration is recorded by the accelerometer. For TOUM, the Cython is placed on the tensed cloth. As the stimulation consists of moving the band in both directions, this change in velocity results in an acceleration that can be measured with the accelerometer, as shown in Fig. 10(b). Sound Pressure Level (SPL) was obtained from the breeze produced by the fan of the AIRM. One DBX RTA-M unidirectional microphone was used and placed perpendicular to the fan opening as shown in Fig. 10(c). The audio was recorded with a sampling rate of 48 kHz. A relation between SPL and voltage was calculated and then, Root Mean Square (RMS) estimation was made using 125 ms windows from the recorded signal.

**Fig. 11:**
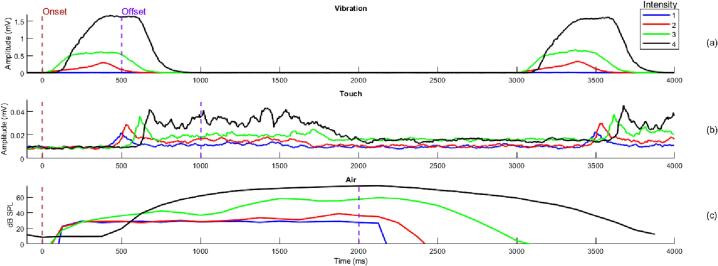
Characterization of the responses from(a) vibration, (b) touch and (c) air modules.

Fig. 11 (a) shows the results after summing the absolute values of all three axles’ recordings from the OpenBCI module while measuring VIBM behavior. The different intensities can be observed easily, as well as the onset latency between the beginning of stimuli (0 s) and when it was first measured. Using the same procedure, Fig. 11 (b) captures how the amplitude increased according to the intensity of the caress and the stimulation onset is delayed proportionally. TOUM starts at 500 ms because the motors have a small delay for activation, it lasts 1000 ms and 500 ms for switch off. Finally, Fig. 11 (c) shows SPLs measured for each stimuli’s intensity. Note that the fan arrives at its maximum point when the stimulus stops at 2000 ms and lasts another 2000 ms to stop. It is also interesting to see that for the first two intensities, the graph shows almost constant behavior. This is due to the presence of noise related to the energization of the fan that disappears while augmenting the intensity level.

### 7.2 EP elicited by NeuroSense

A short experiment was conducted with 10 volunteers. Each subject was exposed to each of the stimuli and intensity 20 times. The exposure consisted on the four intensities randomly permutated with 4 s of intertrial stimuli and posteriorly 6 s of rest. This was repeated 20 times per modality. In total, 240 trials were recorded, 20 per each intensity and modality.

**Fig. 12:**
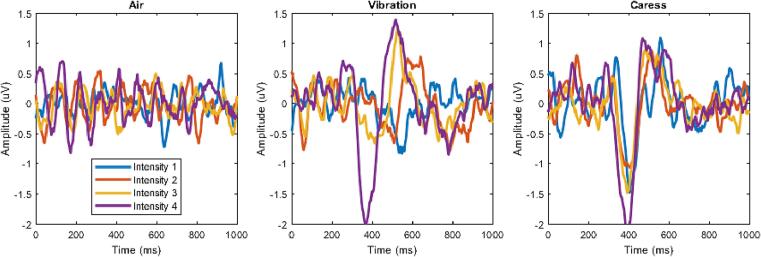
Average ERP waveforms for all subjects at channel C3, displayed from left to right for the modalities of air, vibration, and caress. The various intensities are indicated by different colors.

Trials for each stimulus were extracted for every subject. Subsequently, a study was designed in EEGLAB to extract the grand average ERP across all subjects, modalities, and intensities. Trials were extracted within a time window from −1000 to 3000 ms relative to stimulus onset, with the baseline corrected from −1000 ms to the onset time. The analysis covered a frequency range from 1 Hz to 120 Hz. The obtained ERPs at electrode C3, near the primary somatosensory area, are illustrated in Fig. 12. It is evident that the ERP amplitude varied with the intensity of the stimuli.

Specifically, for vibration and caress stimuli, a clear endogenous modulation in amplitude was observed, indicating a distinct and immediate cortical reaction to these types of tactile input. This modulation in the ERP suggests that the brain can quickly differentiate these stimuli. In contrast, no visible difference in ERP amplitude was observed for air stimulation at any intensity, likely due to the delayed onset latency of the air module. Unlike the rapid and steep activation of vibration and caress stimuli, the activation of the air stimulus increased more gradually.

For the ERP study, time–frequency decomposition was computed using the continuous wavelet transform, and ERDS was extracted using the following formula:

(1) $$ERDS\left(t,f\right)=\frac{P_{task\left(t,f\right)}-P_{baseline\left(f\right)}}{P_{baseline\left(f\right)}}\times 100\%$$

**Fig. 13:**
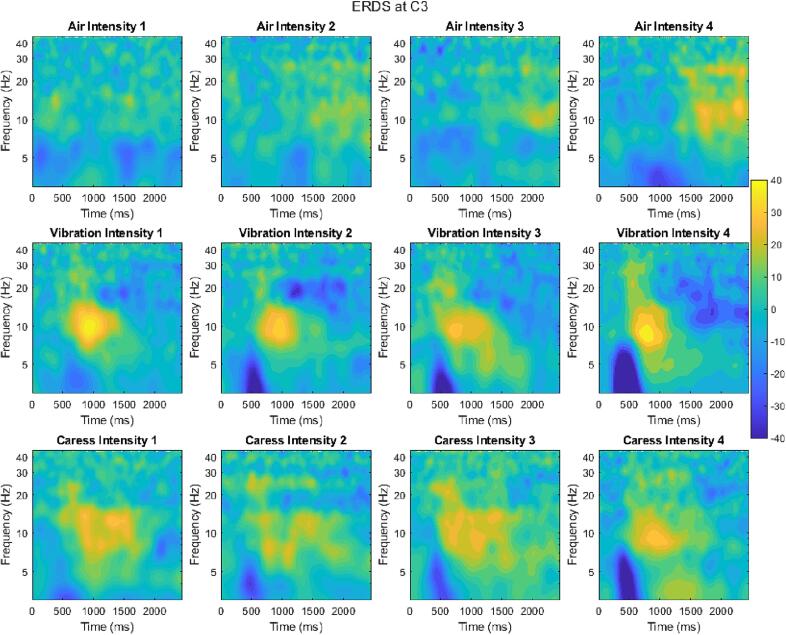
ERDS across all intensities and modalities at the C3 channel. The rows, from top to bottom, represent the modalities: Air, Vibration, Caress. The columns, from left to right, display the intensities in ascending order.

This formula calculates the percentage change in signal power within a specific frequency band during an event-related task compared to a baseline. A positive value indicates event-related synchronization (ERS), and a negative value indicates event-related desynchronization (ERD). Fig. 13 shows the obtained ERDS for the 1 to 50 Hz frequency range. All types of stimulations exhibited an increase in synchronization within the alpha band (near 10 Hz) and the beta band (near 22 Hz), which varied with the intensity of the stimulus.

### 7.3 Limitations and future directions

This study has presented NeuroSense as a tactile stimulator that can reproduce daily life sensations. However, Neurosense presents two important limitations in this study’s results. The first, the non-directly neuropathic pain related evoked stimuli, and second, the habituation attributed to long time stimulation and repetition. NeuroSense evokes three distinct types of sensations that activate A-beta fibers majorly, which are not directly related to neuropathic pain [3]. However, bases on the Gate control model proposed by Malzeck and Wall [24]**,** after stimulating these fibers, interneuron level will release an inhibitor that closes a “*gate”* and, therefore, the pain decreases and eventually disappears [25]. This theory implicitly mentions that A-Beta fibers have an indirect but significant role in the sensation and regulation of pain.

Also, Abraira and Ginty [3] mentioned the importance of LTMR peripheral endings (i.e., A-Beta fibers) and the innervation between LTMR and HTMR for the interpretating the sense of touch and attending allodynia, which may occur after LTMRs become hypersensitive or after there is abnormal signaling in the CNS. Therefore, NeuroSense could particularly be used for studies that evalute the conditions of peripheral nerves indirectly involved in hypersensitivity.

Some recommendations for good practice in matter of evoking EPs are the intensity variability and an adequate resting time after stimuli [4], [26]. In this study, to show the actuators response, a simple paradigm that used several pre-stablished intensities for each type of stimuli and non-variable stimulation and resting times was designed. This should not be the case in paradigms that aim to measure conditions such as allodynia.

For future studies using NeuroSense in healthy subjects, it is recommended to stimulate by using different intensities in random order to avoid habituation and susceptibility [26]**.** Besides, resting time randomness should be included as well during the paradigm structure, and stimulaiton could be applied on different areas of the skin (i.e., three different points of the arm or different sensitive sections of the hand).

### CRediT authorship contribution statement

**Erick A. Gonzalez-Rodriguez:** Writing – original draft, Visualization, Validation, Software, Project administration, Investigation. **Luis Kevin Cepeda-Zapata:** Writing – original draft, Visualization, Validation, Software, Resources, Investigation, Formal analysis. **Angel Antonio Rivas-Silva:** Writing – original draft, Validation, Software, Investigation. **Vania G. Martinez-Gonzalez:** Writing – original draft, Visualization, Investigation. **Luz Maria Alonso-Valerdi:** Writing – review & editing, Supervision, Resources, Methodology, Conceptualization. **David Isaac Ibarra-Zarate:** Writing – review & editing, Supervision, Resources, Methodology.

## Declaration of competing interest

The authors declare that they have no known competing financial interests or personal relationships that could have appeared to influence the work reported in this paper.
